# Improving the user experience of patient versions of clinical guidelines: user testing of a Scottish Intercollegiate Guideline Network (SIGN) patient version

**DOI:** 10.1186/s12913-016-1287-8

**Published:** 2016-02-02

**Authors:** Naomi Fearns, Karen Graham, Gordon Johnston, Duncan Service

**Affiliations:** Healthcare Improvement Scotland, Gyle Square, 1 South Gyle Crescent, Edinburgh, EH12 9EB Scotland UK

**Keywords:** Guidelines, Clinical practice guideline, Patient guidelines, Patient version, Glaucoma, User testing

## Abstract

**Background:**

Guideline producers are increasingly producing versions of guidelines for the public, and knowledge of what the public want from them is also increasing. The aim of this study was to user test a patient version of a SIGN clinical guideline that was designed based on preliminary work for the DECIDE project. SIGN is the leading national guideline producer in Scotland.

**Methods:**

People with a diagnosis of glaucoma and non-professional carers were recruited from across Scotland. User testing was conducted using a think-aloud protocol method. Each session was conducted by one interviewer and one observer. All sessions were recorded and transcribed. The data was analysed, problems with the guideline were identified and resolved and key findings were themed using a user experience model.

**Results:**

Thirteen user testing sessions with people with glaucoma and one with a carer were conducted. Key facilitators of desirability and usability identified include clear branding as a patient version and a clearly described purpose, audience and contents page. Other facilitators include a “friendly” tone which is achieved by the use of colour, quotes, icons, simple language and charts, and brief chunked text.

The value and usefulness of the patient guideline was influenced by its ability to: inform the public, link information to actions, and empower people in their interaction with healthcare professionals. Participants were disappointed by the lack of information on treatment in the patient version, which was outside its scope.

Information on the evidence based guideline production process and the involvement of appropriately skilled professionals was key to the credibility of the guideline. Lack of awareness of guidelines and guideline producing bodies, is a potentially serious threat to findability/accessibility.

**Conclusions:**

It is important for guideline producers to maximise the user experience of the public when they access patient versions of guidelines, particularly given the current low level of access and awareness. One size does not fit all and guideline producers need to strike a balance between keeping the patient version simple and providing sufficient information to facilitate shared decision making and empower the public. Guideline producers may find the results of this study useful in designing their own patient versions.

**Electronic supplementary material:**

The online version of this article (doi:10.1186/s12913-016-1287-8) contains supplementary material, which is available to authorized users.

## Background

Clinical guidelines are tools that are developed systematically to present evidence based recommendations that direct appropriate healthcare [1, 2]. This collation of research, expertise and dissemination of best practice is essential in ensuring patients are treated in a way that is supported by the best evidence available [3]. Patient versions of clinical guidelines, which may help patients to participate more actively in their care and undertake shared decision making, are an area of increasing development and research activity [1].

Many guideline bodies produce patient versions. For example, freely available patient versions are produced in the UK by both the Scottish Intercollegiate Guideline Network (SIGN) and the National Institute for Health and Care Excellence (NICE). Despite this a recent systematic review of the public’s awareness and attitude towards clinical practice guidelines, indicated that awareness of them is low and that the public may not perceive this format of health information any more positively than alternative sources [1]. The review also highlighted the need for guideline producers to make clear how the information contained in the guideline is relevant to the patient and how it can be used in their healthcare. The formatting, clear and unambiguous language, and attractiveness or “glitz” were also identified as important factors [1].

The technique of user testing has been used successfully to evaluate and improve systematic reviews/guideline products and dissemination methods with healthcare professionals [4–6], policy makers [7], and members of the public [8]. However, many questions remain about how best to format and disseminate guidelines, and evidence summaries, to different groups [9].

The aim of this study was to develop and test a SIGN patient guideline, using principles derived from a series of focus groups ([10], Fearns N, Kelly J, Callaghan M, et al: What do patients and the public know about clinical practice guidelines and what do they want from them? A qualitative study, submitted), and a systematic review [1] undertaken by the DECIDE (Developing and Evaluating Communication Strategies to support Informed Decision and practice based on Evidence) project [11]. The findings from this study will inform the design of subsequent SIGN patient versions.

## Methods

### Participants and setting

User testing took place in April 2015, at locations across Scotland including Edinburgh, Glasgow, Stirling, St Andrews and Inverness. Participants were limited to people with a diagnosis of glaucoma or non-professional carers of a person with glaucoma.

Participants were recruited from the SIGN patient network (a database of people interested in SIGN public involvement activities) and from the third sector organisations Carers Scotland and the International Glaucoma Association (IGA). Organisations were contacted by the SIGN patient involvement officer and asked to identify potential participants. Participants were approached by e-mail or phone and provided with a written summary of the research project. We aimed to recruit 12–15 participants to achieve a heterogeneous sample. We considered this likely to be sufficient to reach a saturation of views.

### User testing

User testing was based on a method of data collection and analysis which was developed by Rosenbaum et al. [5, 12]. This method uses a think-aloud protocol and a semi-structured interview guide. User tests took place individually and face-to-face and lasted approximately one hour. One interviewer (KG) conducted all the interviews. A SIGN public partner (a volunteer who works with SIGN to provide a public perspective) acted as an observer (GJ) and took structured notes. A training session was held with the observer and interviewer prior to the start of data collection. The interview guide was structured around the honeycomb model framework of user experience. This has six factors: usability, credibility, usefulness/value, desirability, accessibility and findability [13].

The feasibility of the interview guide was examined in a pilot interview on the 2^nd^ April 2015, and resulted in minor amendments to the interview guide, comprising chiefly of re-ordering of questions. The interview guide is available in Additional file 1.

### The guideline

The user testing examined a draft of the patient version of SIGN guideline 144: Glaucoma referral and safe discharge [14]. SIGN patient versions aim to be an accurate translation of the clinical guideline and only those procedures/interventions recommended in the guideline are included. Further information is only included to help patients and the public understand the recommendations. This draft had been designed based on the principles identified in the earlier work of the DECIDE project [1]. Participants were also shown some supplementary numerical material. All material was presented in hard copy. The full draft guideline is available online in Additional file 2.

### Analysis

The audiotaped interviews were transcribed by one researcher (NF). Phase one of the analysis aimed to identify and prioritise problems that could be resolved and incorporated into the design of the final patient guideline. In this phase the transcripts were analysed by NF and identified barriers were rated for severity. Severity ratings comprised three categories: high (a critical problem that prevents correct use, such as incorrect interpretation or extreme dissatisfaction), medium (a big problem or frustration that slows use), and low (a cosmetic or minor problem) [6]. The interview observer (GJ) used his notes to produce an independent categorisation of identified barriers to guideline use. The researcher (NF) compiled and reconciled the analyses, and grouped them according to the section of the guideline (for example contents page), or topic areas (for example use of colour). Any positive feedback or direct suggestions for improvement were also compiled.

Phase two aimed to identify issues that may be generalisable beyond this specific patient guideline. The findings were refined and analysed thematically using the honeycomb user experience model [13] as a framework. All six factors were examined. However the findability of the SIGN guideline online could not be assessed. The analysis involved re-examining the transcripts and probing the context of the findings. During this phase facilitators of user experience were also identified. The interviewer (KG) and the observer (GJ) examined the findings of this analysis and any conclusions that did not seem authentic to their experience of the user testing were identified; consensus was reached on a final version of the findings.

### Ethics

Ethical approval was obtained from the University of Dundee for a series of focus groups and user testing for the DECIDE project, that informed the development of the patient version tested. Other aspects of this research were undertaken as part of SIGNs service evaluation. Information on the planned data collection for the DECIDE project was provided to the East of Scotland Research Ethics Service and they confirmed that it did not require full ethics committee review under the terms of the Governance Arrangement for Research Ethics Committee (GAfREC) in the UK.

All participants received an information leaflet in advance and gave informed written consent to participate before the session began. The study was discussed and an opportunity to ask questions was given before written consent was taken. The participants were made aware that they could withdraw from the study at any time, that their information would be kept confidential, and that they would remain anonymous in any publications. All participants were refunded for travel expenses and were given a £10 gift voucher.

The names used in quotations are pseudonyms.

## Results

### Participants

Seventeen people were approached and asked to participate. Fourteen people, including the pilot interviewee, agreed to participate (see Table 1). The reasons for non-participation were 1) concern with reading the materials and answering questions “on the spot”, 2) unable to make available dates, and 3) team unable to make contact. All participants were from Scotland and were native English speakers. The mean age of the group was 71 years and nine were male (64 %). The pilot interview was conducted with a SIGN public partner who was a member of the committee responsible for developing SIGN guideline 144 [14]. The pilot interview was transcribed and analysed and did not appear to differ substantially from the other participants contributions, but was removed from the final analysis to avoid bias.

**Table 1:** Participant details

Participant pseudonym	Age	Gender	Level of education	Source recruited from
Alan	66	Male	Higher degree	IGA
Albert	76	Male	Undergraduate degree	IGA
David	77	Male	Higher degree	IGA
Gale	82	Female	Scottish Highers/A-level	IGA
Harry	74	Male	Higher National Certification/Diploma	IGA
James (pilot)	66	Male	Scottish Highers	SIGN Patient Network
Jane	71	Female	Higher degree	IGA
Jennifer	57	Female	Higher degree	Carers Scotland
Mary	76	Female	Higher National Certification/Diploma	IGA
Micheal	71	Male	Undergraduate degree	IGA
Nichola	74	Female	Higher National Certification/Diploma	IGA
Peter	66	Male	Higher degree	SIGN Patient Network
Sam	74	Male	Higher degree	IGA
Stanley	65	Male	Higher National Certification/Diploma	IGA

IGA – International Glaucoma Association

Eleven of the participants were recruited from IGA, two from the SIGN patient network, and one from Carers Scotland. Despite aiming to sample a range of people the group was comprised largely of well educated people, who were likely to have had more knowledge of research and guidelines then the general public. Participants were directly asked about their knowledge of clinical guidelines, four reported that they were knowledgeable to fairly knowledgeable about guidelines (mainly through patient involvement activities and one participant was a retired nurse). The majority (7) had heard of clinical guidelines (largely through the media) but had not accessed one. Three had never heard of clinical guidelines. The participants (aside from the carer) had all been receiving treatment for what they considered to be a long time. The exact number of years was not recorded.

### Findings

A summary of the findings can be found in Table 2.

**Table 2:** Findings

Theme	Findings
Usefulness/Value	• Patient versions of guidelines can inform and empower people to ask questions. • They can help people to anticipate what to expect when seeing a healthcare professional or having an intervention. • They may be most useful to patients around the time of their diagnosis. • Information about risks is most useful if directly associated with information about self management or any form of action. • Simple diagrams and charts can communicate information clearly. • It is helpful to flag clearly any important areas not covered by the guideline. • Signposting to organisations that can provide help and further information is valued.
Usability	• Language should be kept as simple as possible • User testing may help to identify how much technical information to include. • Small font size, use of light/pale colours, and too much material on a page were major barriers to use of the guideline by this patient group. • Clear flagging of recommendations using headings/icons works well. • A risk of 2 in 100 was interpreted by some as very high and others as very low. • Icons for levels of recommendation worked best when kept recognisable, with a clear link to the intended message. • Vague or generic icons can cause confusion and be misinterpreted e.g. a blue circle can be interpreted as a zero. • Uncertainty was effectively communicated by the “?” icon but people may not know how to respond to this information.
Credibility	• Credibility arose from information on the guideline production process, and the involvement of qualified professionals. • The status of the guideline is important (do health services recognise the recommendations). • Credibility may be threatened by pathways or recommendations that do not fit with the patient’s own experiences.
Desirability	• Participants were very positive about the look and feel of this patient version. • Aspects that increased desirability included a friendly tone, simple language, chunking of text, the use of colour, glossy “high quality” look, and use of icons/images. • A friendly feel is achieved by informal language, use of colour, and the inclusion of quotes and images/icons. • Negative language or images, and a bureaucratic/dogmatic tone were disliked. • Quotes can personalise the material, giving it an engaging and friendly tone, and emphasising a particular message.
Accessibility/Findability	• The brief contents page, with simple question based headings was clear and facilitated flicking to relevant sections. • The participants were very concerned about the apparent lack of dissemination of patient versions of guidelines. • It is important for printed copies of the guideline to be available. • The patient version must be tailored to the intended audience’s needs (e.g. font size, language/numerical information). • Information on how to access the services/interventions recommended is important. • Clear branding as a patient version is required. • Clear information on “who this booklet is for” encouraged people to read and share the guideline. • It is important to give telephones numbers and addresses as well as websites for signposted organisations.

### Usefulness and value

There was a strong consensus amongst the participants that the patient guideline was very useful, many expressed regret that they had not had access to something like it earlier. The most important time to have access to this type of information was identified as around the time of diagnosis.

*“If my optician at the time…had given me…this booklet…it would have made things so much easier for me. Its one thing not knowing but ignorance is not always bliss as it turned out.”* [Stanley]

Information in the guideline was considered useful even if it was simply providing “*background information*” that the participant was unable to identify a direct use for. An example of this was the information provided on types of glaucoma, which was new information for many of the participants, despite having had glaucoma for a long period of time.

The guideline can be empowering by giving patients knowledge about what to expect when accessing health services, providing sufficient information to ask further questions or to request more information on a specific intervention.

*“I think it would give you guidelines to actually make sure you were being given what is an appropriate standard of care.”* [Mary]

Aspects of this guideline that participants highlighted as useful included: information on their health condition, risk factors, medical tests, self management, the referral and discharge pathway and what to expect from healthcare professionals at different stages, and signposting to other organisations.

Participants considered risk information to be most useful when linked to actions that can be taken to counter the risks, in this case regular eye checks. If this direct link is not made some consider this information to be “*scary*” and unhelpful.

*“Well, it [supplementary material on cardiovascular risk] certainly puts the information across. It might frighten a few folks into having a heart attack [laughs]…but what would they do about it?”* [David]

This patient guideline did not cover treatment for glaucoma because it was based on SIGN guideline 144: Glaucoma referral and safe discharge. Despite this being flagged on page 3 of the guideline many participants raised the lack of treatment information. Those that did realise that the guideline did not seek to cover treatment were surprised that something this important had been left out.

Outlining who the guideline is for, through clear sub titles on the front cover and a “Why have I been given this booklet” page was useful and encouraged people to “*pick up*”, read, and share the guideline with others. The draft guideline is available in Additional file 2.

Some concerns were expressed about the “Why have I been given this booklet” page being overly prescriptive in tone. For example it did not acknowledge carers or friends as people who may be interested in the material.

### Usability

Some major barriers to usability were identified including the use of too small a font size, colours that were too light and pale to be read, and too much material on some pages. While the level of difficulty caused by these problems is likely to be particularly high in this patient group the preference for chunking [15] (for example bullet points and images to break up the text), and limited material on each page has also been identified in our earlier work ([1], Fearns N, Kelly J, Callaghan M, et al: What do patients and the public know about clinical practice guidelines and what do they want from them? A qualitative study, submitted). The participants also highlighted the need to work with people’s pre-existent associations with colour e.g. red stop and green go.

**Fig. 1:**
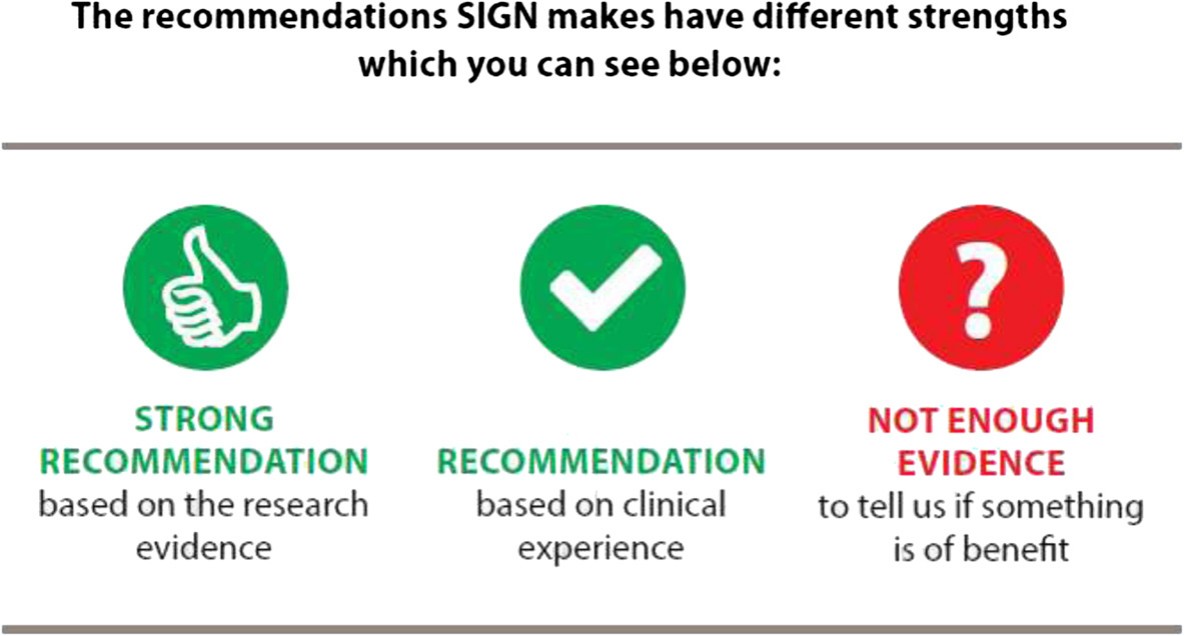
Recommendation level icons

The use of a set of simple icons to communicate the level of evidence underlying recommendations was tested in this patient version (see Fig. 1). The icons incorporated a text description of the level of evidence. The response to these simple icons was positive, they were recognisable and clear, providing an instant message on the level of the recommendation.

*“…the recommendation thing, strong medium and not enough evidence kind of thing that’s a very useful marker…knowing how the experts marked it that in itself was helpful to me.”* [Stanley]

However, the reason for having two levels of positive recommendation was not clear, despite providing an explanation on the third page of the guideline. It was generally interpreted as meaning that the recommended level was less important than the strongly recommended level, without picking up that one is based on research evidence and one on clinical experience. While the question mark symbol was effective in communicating uncertainty, many people did not respond positively to the idea of uncertainty in the evidence base. The participant’s key difficulty was not knowing how to respond to this information, essentially they were left wondering what the point of the “not enough evidence” recommendation was.

*“No [I wouldn’t follow this recommendation], and I wouldn’t worry about it either because there is not enough evidence.”* [Jennifer]

It appeared that not enough contextual information has been provided with the “not enough evidence” recommendation and this may have compounded the negative response to it.

**Fig. 2:**
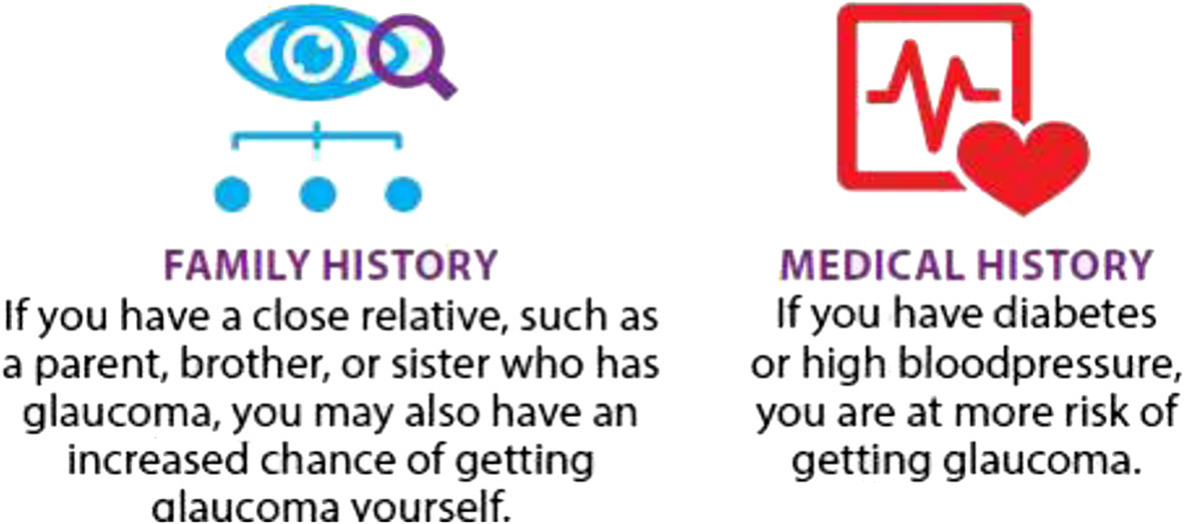
Risk information

The use of icons throughout the booklet helped to communicate information quickly and clearly, as well as breaking up the page, highlighting key points and giving a friendly look to the guideline (see Fig. 2). The use of icons and a one sentence text description to convey risks was received particularly positively.

*“I have normally only seen this set out as a table. This is that wee bit friendlier....You have said no more than you have to it is kept very simple and yet it says everything you need to…you read it and the thing just makes sense.”* [Stanley]

It is important to ensure that any icons used relate clearly and directly to the message. One piece of supplementary material shown to the group used a blue circle to represent diabetes; this was interpreted as a zero and taken to mean zero risk by a participant, the opposite of the message that was intended.

**Fig. 3:**
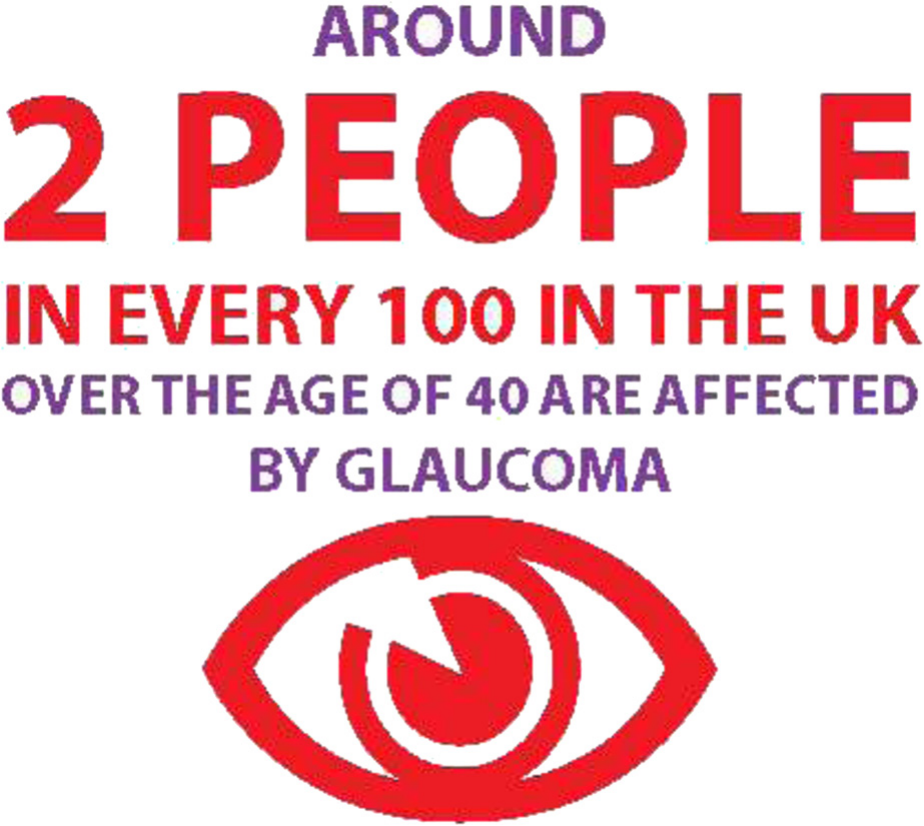
Supplementary numerical information

No numerical information was included in the patient version so some was presented as supplementary material (see Fig. 3). Participants interpreted a risk of 2 in 100 for developing glaucoma in widely different ways. Some considered this a surprisingly high number and some a very low one and it was clear that the information was not sufficient for people to interpret the numbers clearly.

Participants highlighted some lengthy or repetitive sentences, and sections with a bureaucratic tone. However, the use of language was praised throughout the guideline for being simple and understandable.

*“It wasn’t way above people’s heads and…particularly medical type of things that can be something that is too easily done.”* [David]

The description of diagnostic tests for glaucoma was overly technical for most participants (see Additional file 2), due to the inclusion of terminology like “*slit-lamp biomicroscopy*”. Nonetheless, some participants did appreciate the inclusion of this technical information.

Participants were shown bar and pie charts from the supplementary material (see Additional file 3). Simple graphical aids were useful in understanding numerical information – the simpler the better. Those who expressed a preference for pie charts felt they were easily interpreted and instantly understandable. While those who expressed a preference for bar charts suggested they could provide more information and allow for more comparisons, although they can be harder to interpret.

### Credibility

Based on preliminary user testing, information on SIGN and the guideline production process was contained at the back of the guideline. The information reassured participants about the guidelines credibility. It was important to participants to know that the guideline was “*scientific*”, meaning evidence based. For some the involvement of qualified professionals in its production was a key element of credibility which appeared more meaningful than detail on the guideline production process.

*“…sometimes people are a bit suspicious about some of the healthcare advice that you are given…you want to know how scientific it is…how accurate it is…from this I am sure that…it is scientific and accurate…”* [Michael]

Another element of interest to participants was the status of the guideline, that is can it be used to verify what standard of care they should expect and as leverage for accessing their preferred interventions?

*“who has made them, what is the kind of professional background of the people who have done them and how can I use them. Can I use them to force them [health services]?”* [Jennifer]

A possible threat to credibility arose from pathways and recommendations that did not fit with the person’s own experience as a patient. An example of this was the assumption that people are referred to an eye specialist by their optometrist when some of the participants had been referred by their GP.

### Desirability

There was strong praise for the look and design of the guideline. Aspects that made the guideline particularly desirable included; a friendly tone and simple language, the use of colour, chunking of text, use of icons/images, a glossy “high quality” look, and clear branding as a patient/public version.

Quotes were beneficial in personalising/humanising the material, and giving it a friendlier feel. They engaged the participants, upping the interest level in the material and thereby emphasising a particular point.

*“… they do tend to personalise it…you are more likely to take something on board if a real human being has said it…“if I had known about this earlier I would have done something about it”…then you think “oh I had better do something about it…”* [Peter]

The inclusion of quotes did not add value for the more factually orientated participants, but they recognised that they could be helpful for others. Participants highlighted that quotes could have unintended or unclear messages, for example the use of a quote about having to stop driving after diagnosis may put people off having their eyes tested.

Negative images were highly undesirable. The cover image (a pair of hands holding glasses) was described as “*helpless*” and ”*anxious*” in emotional tone. It was also disliked for its indirect relationship with glaucoma and was considered potentially misleading since it could be identified with opticians’ general services instead of glaucoma. Participants were strongly in favour of a cover image that communicated glaucoma as clearly as possible.

For some “scary” language such as use of the word “blindness” was also undesirable and off putting, while this was balanced by others emphasis on getting the message that your eye sight is at risk, unless you have regular eye checks, across clearly.

### Accessibility and findability

Dissemination was a key concern of the participants:

“*I hope it [the guideline] actually gets through to the people*” [Sam]

People’s lack of knowledge about the roles and functions of SIGN, and the SIGN logo not being recognisable or clearly identifying SIGNs role in guideline production, led to increased concerns about dissemination.

“ *…have they heard of SIGN? You know for the average punter. Do they know what SIGN is? Maybe there is a separate thing of promoting SIGN and saying what it is, publicity wise*.” [Peter]

Participants were eager to highlight that having guidelines and other materials for patients available on the internet was not sufficient to disseminate the guideline. Printed copies should also be distributed in public buildings, and phone numbers and addreses provided for signposted organisations.

As noted above considering the needs of your intended audience is key to accessibility. The text size and use of colour in the initial version of this guideline was not appropriate for people with visual impairment. Considering the needs of the intended audience in regard to complexity of material presented and technical language is important.

A strong emphasis was placed on letting people know that eye tests are free in Scotland, as a method of encouraging people to access them. This illustrated the need to include information on how the public will access the interventions recommended in a patient version.

### Implementing the user testing findings

Many of the findings pointed to clear solutions which were implemented, for example: changing the cover image, increasing font size, using darker colours, reducing the amount of information on each page. A table listing the changes made to the draft guideline is available as Additional file 4. The final patient version which incorporated the results of this user testing is available on the SIGN website [16].

## Discussion

A patient version of SIGN guideline 144 was designed which incorporated findings from the work of the DECIDE project ([1, 10], Fearns N, Kelly J, Callaghan M, et al: What do patients and the public know about clinical practice guidelines and what do they want from them? A qualitative study, submitted). This study suggested that the patient version met many of the participants needs. Key barriers to use of the guideline for this patient group included insufficient font size, pale colours, and too much information on each page – making the document inaccessible for people with visual impairment. These aspects were all easily addressed in the final version [16]. The importance of user testing of materials developed for the public is clear, since these major barriers may not otherwise have been addressed.

Key facilitators of desirability and usability identified include clear branding as a patient version and a well described purpose and contents page. Only around half of published patient versions in the English language currently do this [Santesso N, Morgano GP, Jack S, et al: Communicating clinical practice guidelines to patients and the public: A content analysis of patient versions, submitted]. Other important facilitators include a “friendly” clear tone which is achieved by the use of colour, quotes, icons, simple language and charts, and brief chunked text.

The value and usefulness of the patient guideline was determined by its ability to: inform the public, link information to actions, and empower patients and carers in their interaction with healthcare professionals. Participants were disappointed by the lack of information on treatment in the patient version, which is a result of the scope of SIGN guideline 144. Guideline producers may need to strike a balance between generating additional information for patient versions and sticking to the scope of the full guideline (and potentially not giving the public what they want). Achieving this balance is discussed in the Guidelines International Network (GIN) toolkit [17]. It is important to make clear what is, and what is not covered, from the front page onwards, to avoid disappointment and confusion in the public.

In the patient version examined, “strongly recommended” indicated a recommendation based on research evidence and “recommended” indicated a recommendation based on clinical experience. While participants clearly understood that there were two levels of recommendation and that the ‘thumbs up’ symbol was a stronger endorsement than the ‘tick’ symbol, they generally did not connect this with the underlying evidence. It is not clear whether this is to do with a lack of awareness of what constitutes research evidence, or a failure to communicate it clearly in this patient guideline. Recent research examining the public’s understanding of plain language summaries produced by the Cochrane collaboration indicated that less than 50 % of participants correctly interpreted information about the quality of evidence [18]. It may also reflect the participants own emphasis on the involvement of expert healthcare professionals in giving credibility to the guideline, perhaps indicating that the distinction between expert opinion and research evidence is relatively unimportant to some of the public.

The shock expressed by members of the public at the idea of interventions being in use while the evidence underlying them is uncertain may also reflect the lack of awareness of varying levels of research quality in much of the general public [1]. Guideline producers are faced with the challenge of deciding how much information to provide that attempts to explain research concepts. Similarly they must decide how much information to provide on the methodology of guideline production. Users of the guideline may become frustrated, particularly if excessive information delays access to the key messages of the patient version [Fearns N, Kelly J, Callaghan M, et al: What do patients and the public know about clinical practice guidelines and what do they want from them? A qualitative study, submitted]. Layering information so that readers can drill down for more detail may provide an answer to this in electronic formats [19] but it is more challenging in print products.

Information on the evidence based production process and the involvement of expert healthcare professionals was key to the credibility of the guideline. Patients and the public do want information on the guideline production process to be included in the patient version. The position and extent of this information may need to be determined on a guideline by guideline basis by considering the needs of the likely audience.

There is considerable heterogeneity in people’s preferences for the provision of technical information/evidence, the use of language, the inclusion of numerical information, patient stories/quotes and so on. It is clear that materials must be tailored to the audience and the individual as much as possible. Electronic media provide clear methods of layering and personalisation [19]. Some of the advantages of layering were retained in this paper based patient version by the use of a clear contents page that facilitates flicking to the desired sections, creative use of formatting (for example the use of icons, boxes, and tables) and careful consideration of the order of presentation of information. Another approach to meeting the needs of different groups is the creation of multiple versions, with varying complexity of contents. Accessibility is also facilitated by making paper and electronic copies available.

The purpose of the “not enough evidence” form of recommendation may be made more relevant to the general public and patients when it can be linked directly to patients own preferences and values through a choice of actions for the patient, for example via decision making tools or self-management activities. The need for patient versions of guidelines to focus on self management and facilitate decision making is a clear theme from the work of the DECIDE project, and it appears that this is not currently being incorporated into all English language patient versions [Santesso N, Morgano GP, Jack S, et al: Communicating clinical practice guidelines to patients and the public: A content analysis of patient versions, submitted]. It remains uncertain how best to facilitate decision making, but there are various ways in which options can be presented, including a plain language summary [8], interactive summary of findings tables [6] and option grids [20].

Participants responded very positively to the simple risk information presented using icons in the patient version we tested. However there is some evidence that people’s understanding of risks is aided by the provision of numbers as well as words [18, 21, 22]. Adding natural frequencies, to the simple form of risk information presented in the glaucoma patient guideline and examining its usability would seem a natural progression in future SIGN patient versions.

The presentation of risks in absolute and relative terms is necessary to maximise understanding in consumers and clinicians [23]. User testing made plain that the public’s interpretation of 2 in 100 varied widely and that contextual information, such as the actual number of people in the UK with glaucoma (at a given point) was required to aid the interpretation of this information.

### Strengths and weaknesses

A strength of this study was the use of a method of user testing that has proven effective in obtaining practical and transferable findings, in a group of participants who were all drawn from members of the potential audience of the patient version. It was extremely valuable to receive input into the design of the interview schedule, the data collection and analysis of the data from a SIGN public partner.

While efforts were made to recruit heterogeneous group of participants, this was only partially successful. Only one carer participated and more men were recruited (64 %) than women. However, there were no notable differences between the responses of male and female participants. The group was also relatively highly educated and may have a greater knowledge and understanding of research and clinical guidelines than the general public. Even so, most of the group were not aware of clinical guidelines, and had not seen a patient version before.

During the iterative analysis and data gathering process it became clear that no new themes were arising with subsequent interviews, suggesting that saturation may have been achieved within the group of participants that the recruitment process was capable of accessing. The transferability of the findings of this research to different socio-cultural settings is limited, although some transferability is credible. For example, some of the findings from this study might not apply to a guideline developed for young people, or in another country. It should also be noted that the patient version was developed following a set of preliminary work with people with a range of health conditions and backgrounds [Fearns N, Kelly J, Callaghan M, et al: What do patients and the public know about clinical practice guidelines and what do they want from them? A qualitative study, submitted].

The glaucoma guideline that was examined in this study did not cover treatment and this may limit the scope of the findings. Additional material was used to examine participant’s response to information about treatment. A possible avenue of future research would be the user testing of a variety of patient versions of future SIGN guidelines, which would address this limitation and help to establish the transferability of the findings of this study.

## Conclusion

There is currently a low level of access and awareness of patient versions of clinical guidelines [1]. Guideline producers should examine the findings of this study and the earlier work of this project ([1, 10], Fearns N, Kelly J, Callaghan M, et al: What do patients and the public know about clinical practice guidelines and what do they want from them? A qualitative study, submitted) to maximise the user experience for members of the public when they do access a patient version. “*One size does not fit all*” and guideline producers need to strike a balance between keeping the patient version simple and providing sufficient information to facilitate shared decision making and empower the public. A friendly and simple look and feel appears highly important in making a desirable and usable patient version.

The findings from this study helped to support the update of the Guideline International Network Toolkit Chapter 4 on developing patient versions of guidelines [17].

## Additional files

Additional file 1: **Interview Guide.** (PDF 311 kb)

Additional file 2: **Draft patient version of SIGN glaucoma guideline.** Permission is given to reproduce this document. (PDF 556 kb)

Additional file 3: **Supplementary bar and pie charts.** (PDF 144 kb)

Additional file 4: **Table of changes made to the draft SIGN guideline following user testing.** (PDF 283 kb)

## Abbreviations

DECIDE: Developing and Evaluating Communication strategies to support Informed Decision and practice based on Evidence
GAfREC: Governance Arrangement for Research Ethics Committee
GIN: Guidelines International Network
NICE: National Institute of Clinical Excellence
SIGN: Scottish Intercollegiate Guidelines Network
