# Supplementary material for: Improving the user experience of patient versions of clinical guidelines: user testing of a Scottish Intercollegiate Guideline Network (SIGN) patient version
Source: BMC Health Serv Res. 2016 Feb 2;16:37. doi: 10.1186/s12913-016-1287-8 (PMC4736267; doi:10.1186/s12913-016-1287-8)
Supplement: Supplementary file 1 — Interview Guide. (PDF 311 kb) [file 12913_2016_1287_MOESM1_ESM.pdf]

USER TEST INTERVIEW GUIDE

**DECIDE WP3 User testing interview guide:**<br>**Glaucoma PCGL**	
**Test person no.:**	
**Place:**	
**Date:**	
**Interviewer/notetaker:**	

1. Checklist

For facilitator, bring:

- Printed copy of the glaucoma guideline and any additional materials.
- Participant information sheet and consent form
- Voucher
- Travel expense claim information/forms

For observer/note taker, bring:

- Paper to take notes (or laptop with a copy of this interview guide, one for each session).
- Tape recorder

2. Introduction

> *Go through the* written information they have already received

- What we are doing
- Who is participating, why we invited you
- How the test is conducted
- What happens to the data/recording
- Rights to quit or retract recording
- Questions?
- Sign consent form
- Thank you for agreeing to participate

> Turn on audiorecorder.

Background questions

A	**Ask:** What is your date of birth?

B	**Ask:** What is your level of education?
- None - School (for example GSCEs, Standard level) - High school – (For example Scottish higher, A-levels - Bachelor's degree (for example: BA, BSC) - Master's degree (for example: MA, MSc) - Doctorate degree (for example: PhD)	
Other:	

C	**Ask:** Record gender

D	**Ask: Have you ever heard of a clinical guideline?**<br>**Ask: Have you ever heard of a patient clinical guideline?**
	If not: explain what one is and that we will be asking them to look at one on Glaucoma today.

Repeat instructions

A short bit of repetition before we begin.

No right or wrong answer
You are not being tested, it is our material we are testing. There are no right or wrong answers to our questions. If you think something is easy or difficult, clear or confusing, if you understand or don't understand, we just want to know about it.

Think out loud
Think out loud. Tell me what you are thinking, what you see, what you find confusing or surprising, even the least little bit. For instance:

- What you are looking at, describe your experience of it.
- If you are unsure about anything
- If you are surprised by anything
- If there are things you don't understand, just say so.

My role
My role is to ask questions. But, since it is your opinion we are interested in, I will be otherwise saying as little as possible. You will have another opportunity to ask questions about what we are doing after we do the user testing.

1	**Present with SIGN glaucoma booklet**<br>Say: please have a look and tell me what you think of it.
1a	**What are your first impressions?**
1b	**If you saw this would you pick it up? What do you think of the way it looks?**
1c	**Do you know what this is? Do you know who this is for?**
1d	**How do you find the title?**

2	**Present alternative cover 1**<br>Say: please have a look and tell me what you think of it.
2a	**What are your first impressions?**
2b	**If you saw this would you pick it up? What do you think of the way it looks?**

1c	**Do you know what this is? Do you know who this is for?**

3	**Present alternative cover 2**<br>Say: please have a look and tell me what you think of it.
3a	**What are your first impressions?**
3b	**If you saw this would you pick it up? What do you think of the way it looks?**
3c	**Do you know what this is? Do you know who this is for?**

4	**Present with contents page only (pg1).**<br>Say: please have a look at just the contents page and tell me what you think of it.
4a	**Does this tell you what you want to know about what is in the guideline?**
4b	**Is it clear?**

5	**Present with 'why have I been given this booklet' (pg2).**<br>Say: please have a look and tell me what you think of these pages.
5a	**Is this information clear?**
5b	**Is it helpful?**

6	**Present with 'what is this booklet about' (pg3).**<br>Say: please have a look and tell me what you think of these pages.
6a	**Is this information clear?**
6b	**Is it helpful?**

7	**Present with 'increased eye pressure' example recommendations based on evidence (pg9).**<br>Say: please have a look at this page and tell me what you think of it.
7a	**Do you understand this recommendation?**
7b	**Would you do what is recommended?**
7c	**What do you think of the symbol?**
7d	**Would you add or remove anything?**

8	**Present with recommendation based on clinical practice 'close relative has glaucoma' (pg9).**<br>Say: please have a look at this page and tell me what you think of it.
8a	**Do you understand this recommendation?**
8b	**Would you do what is recommended?**
8c	**What do you think of the symbol?**
8d	**Would you add or remove anything?**

9	**Present with 'not enough evidence' point 'should I have a patient-held record'? (pg9).**<br>Say: please have a look at this page and tell me what you think of it.
9a	**Do you understand this recommendation?**
9b	**Would you do what is recommended?**
9c	**What do you think of the symbol?**
9d	**Would you add or remove anything?**

10	**Still using pg9 recommendations.**
10a	**Do you understand how these recommendations are different to each other?**

11	**Present with 'other tests' (pg12).**<br>Say: please have a look at this page and tell me what you think of it.
11a	**What do you think about these recommendations?**
11b	**How would you use the information on this page?**
11c	**Would you add or remove anything?**

12	**Present with ‘referral to hospital eye care’ (pg13).**<br>Say: please have a look at this page and tell me what you think of it.
12a	**What do you think about these recommendations?**
12b	**How would you use the information on this page?**
12c	**Would you add or remove anything?**

13	**Present with example diagram (pg4)**<br>Say: please have a look at these diagrams and tell me what you think of them.
13a	**Is it useful?**
13b	**Is it clear?**
13c	**Would you change anything about the diagram?**

14	**Present with ‘what are the risks for glaucoma’ (pg7 – risks without numbers)**<br>Say: please have a look at this information and tell me what you think of it.
14a	**Is it useful?**
14b	**Is it clear?**

15	**Present with ‘statistic 1’ (risks with numbers)**<br>Say: please have a look at this information and tell me what you think of it.
15a	**Is it useful?**
15b	**Is it clear?**

16	**Present with ‘statistics 2’ (risks with numbers)**<br>Say: please have a look at this information and tell me what you think of it.
16a	**Is it useful?**
16b	**Is it clear?**

17a	**Now with both ‘statistics 1’ or ‘statistics 2’.**
	**Do you prefer either of them? Why?**

18	**Present with an information only page (pg6)**<br>Say: please have a look at this information and tell me what you think of it.
18a	**How would you use this information?**
18b	**Do you like the way it looks?**
18c	**Would you add or remove anything?**

19	**Present with a page with personal story on it (pg7).**<br>Say: You will have seen some personal stories throughout the guideline, how did you find them? Have another look at some others if it helps.
19a	**What do you think of the personal stories?**
19b	**Why?**

20	**Present with 'what is SIGN' page (pg20).**<br>Say: please have a look at this information and tell me what you think of it.
20a	**How would you use this information?**
20b	**Would you add or remove anything from this information?**

21	**Say: Think about the guideline as a whole. Have another look through it if you want.**
21a	**Does it look appealing? Would you pick one up?**
21b	**Would you add anything to or remove any of the information?**
21c	**How do you find the language in it? Would you change it at all?**
21e	**Would you use the advice and information in this guideline? Why?**
21f	**Is there anything missing from it that you would like to know about?**

21g	**Is there anything you would changes about the guideline?**

22	**Present with ‘statistics 3’ – Pie & bar charts. Flag up stroke information.**<br>**Say: please have a look at these graphs and tell me what you think of them**
22a	**How would you use this information if it was given to you?**
22b	**Are they clear?**
22c	**Which do you prefer?**

23	**Present with ‘risks’ CVD page – flag up on cardiovascular risks.**<br>Say: please have a look at this information and tell me what you think of it.
23a	**Is it useful?**
23b	**Is it clear?**

24	**Say: Thank you very much – that’s all. Anything else to add.**
