# Supplementary material for: Improving the user experience of patient versions of clinical guidelines: user testing of a Scottish Intercollegiate Guideline Network (SIGN) patient version
Source: BMC Health Serv Res. 2016 Feb 2;16:37. doi: 10.1186/s12913-016-1287-8 (PMC4736267; doi:10.1186/s12913-016-1287-8)
Supplement: Supplementary file 2 — Draft patient version of SIGN glaucoma guideline. Permission is given to reproduce this document. (PDF 556 kb) [file 12913_2016_1287_MOESM2_ESM.pdf]

Healthcare Improvement Scotland

GLAUCOMA

What does the SIGN guideline say?

A BOOKLET FOR PATIENTS, THEIR FAMILIES AND CARERS

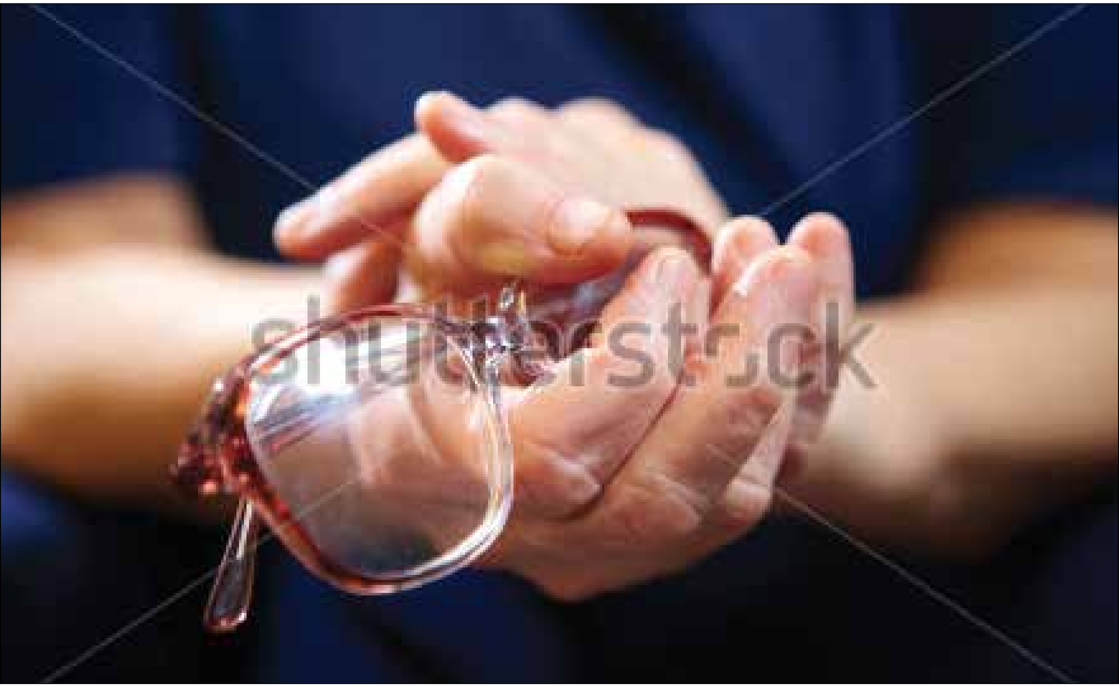

SIGN

CONTENTS

WHY HAVE I BEEN GIVEN THIS BOOKLET?
2

WHAT IS THIS BOOKLET ABOUT?
3

WHAT IS GLAUCOMA?
6

WHAT ARE THE RISK FACTORS FOR GLAUCOMA?
7

WHAT CAN I DO TO HELP MYSELF?
8

HOW WILL I KNOW IF I HAVE GLAUCOMA?
10

REFERRAL TO HOSPITAL EYE CARE
13

HOSPITAL EYE CARE
15

DISCHARGE FROM HOSPITAL EYE CARE
16

WHERE CAN I GET MORE HELP?
19

WHAT IS SIGN?
20

WHY HAVE I BEEN GIVEN THIS BOOKLET?

You have been given this booklet because:

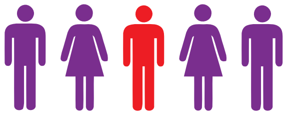

YOU HAVE A HIGH RISK OF GETTING GLAUCOMA

SEE PAGE X

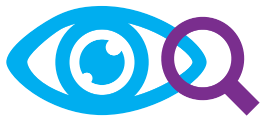

YOUR OPTOMETRIST THINKS YOU MIGHT HAVE GLAUCOMA

SEE PAGE X

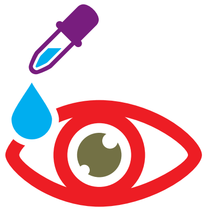

YOU HAVE GLAUCOMA AND ARE BEING LOOKED AFTER BY YOUR OPTOMETRIST OR HOSPITAL EYE SPECIALIST

SEE PAGE X

This booklet explains:

Risk factors for glaucoma **(see page 7)**
Examinations and assessments **(see page 10)**;
Referral to hospital eye care services **(see page 13)**
Discharge from hospital eye care services **(see page 16)**

WHAT IS THIS BOOKLET ABOUT?

This booklet aims to explain the recommendations in a clinical guideline from the Scottish Intercollegiate Guidelines Network (SIGN) clinical guideline on glaucoma referral and safe discharge. The guideline is based on what we know from current medical research. More about SIGN and how guidelines are produced can be found on page 20.

This booklet also gives advice based on the considered opinion of healthcare professionals who are trained to check your eyesight and look for signs of eye disease.

The recommendations SIGN makes have different strengths which you can see below:

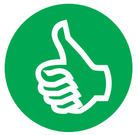

STRONG RECOMMENDATION
based on the research evidence

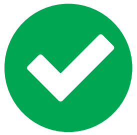

RECOMMENDATION
based on clinical experience

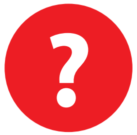

NOT ENOUGH EVIDENCE
to tell us if something is of benefit

This booklet gives you information about the care you are likely to get but does not have a lot of information on glaucoma itself. The booklet does not give advice on the treatment of raised eye pressure (ocular hypertension) or glaucoma. **On page 19** there are details of organisations who can give you information about glaucoma.

If you would like to see the clinical guideline, please visit **www.sign.ac.uk.**

Normal Eye

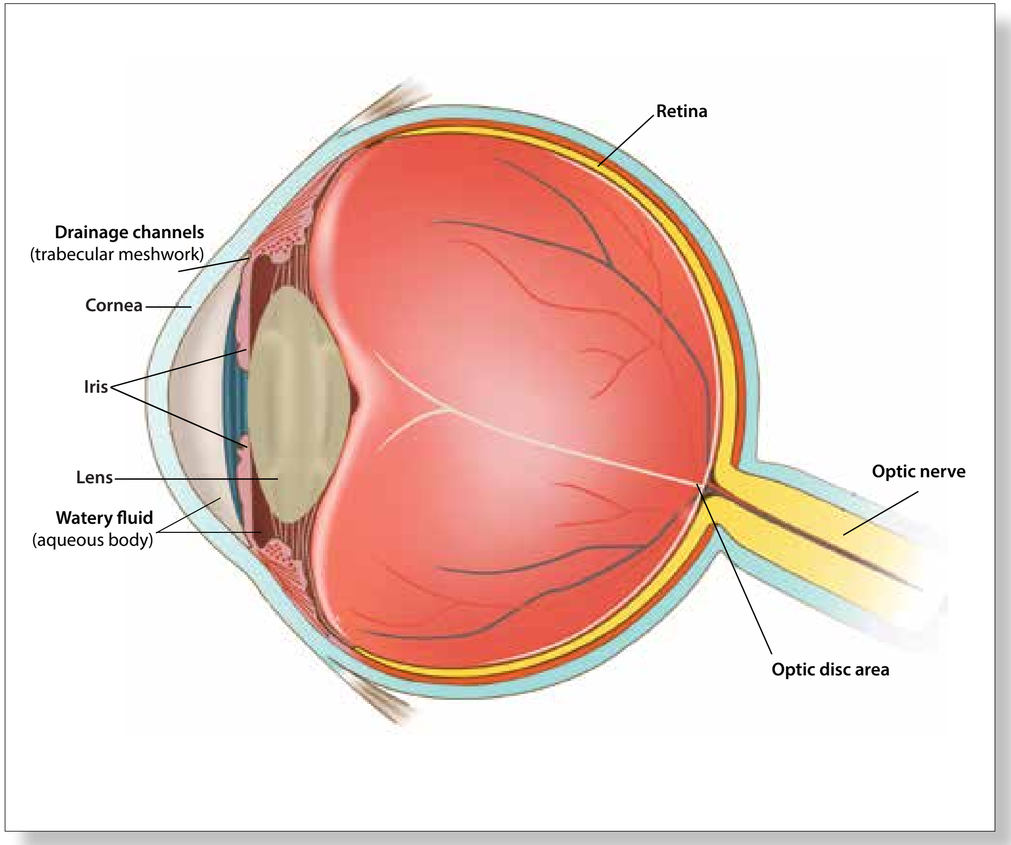

GLAUCOMA: what does the SIGN guideline say?

YOUR EYE

Your eye needs a certain amount of pressure inside it to keep a proper eyeball shape so you can see. If you have too much pressure in your eye, the optic nerve becomes damaged. Some people have normal eye pressure but damage to the optic nerve can still develop.

The watery fluid in your eye passes through your pupil into the space in front of your iris. It leaves your eye through tiny drainage channels called trabecular meshwork. These drainage channels are in the space between the front of your eye (the cornea) and your iris, and they return the fluid to the blood stream. Normally, the amount of fluid produced is balanced by the fluid draining out. If the fluid cannot drain properly, or if too much is produced, then your eye pressure will rise. The name given to increased eye pressure is ocular hypertension. Ocular hypertension can increase your risk of getting glaucoma.

Eye pressure

Eye pressure is measured in millimetres of mercury (mm Hg), the same unit of measurement used in measuring your blood pressure. Normal eye pressure ranges from 12-25 mm Hg. Your optometrist may wish to repeat pressure measurements if your measurement is at the higher end of the range.

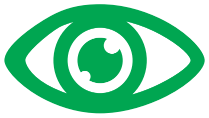

NORMAL
EYE PRESSURE RANGES FROM 12-25 MM HG

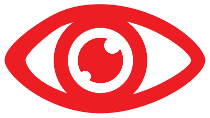

HIGHER
EYE PRESSURE IS ANYTHING MORE THAN 22 MM HG

WHAT IS GLAUCOMA?

Glaucoma is the name for a group of eye conditions that involve damage to the optic nerve in your eye. It is the leading cause of blindness worldwide. In the UK, glaucoma is the second most common cause of sight loss.

The risk of glaucoma increases as you get older so it is important for you to get your eyes tested regularly. If glaucoma is not treated it can cause you to suffer sight loss and a small number of people eventually go blind. If it is diagnosed and treated early enough, further damage to your sight can be prevented. Usually, people have to have treatment for glaucoma for the rest of their lives.

Types of Glaucoma

OPEN ANGLE GLAUCOMA

There are different types of glaucoma but the most common one is chronic open angle glaucoma. 'Angle' refers to the drainage area in your eye from where fluid leaves it via channels. (See page#). In open angle glaucoma, the drainage channels within your eye become slightly blocked, stopping fluid from draining properly.

This type of glaucoma develops slowly. People who have this also have increased pressure in their eye. Some people however will have glaucoma even with normal eye pressure.

ANGLE CLOSURE GLAUCOMA

This type of glaucoma happens when your iris has moved forward causing your drainage angle to close. Since the fluid cannot escape from your eye, there is a sudden and painful build up of pressure in your eye.
This type of glaucoma is much less common.

SECONDARY GLAUCOMA

This type of glaucoma can be open angle or closed angle. It may happen because of an eye injury or another eye condition.

DEVELOPMENTAL GLAUCOMA

Developmental glaucoma happens when the eye does not form properly. It may be there at birth or develop shortly after birth. This type of glaucoma is rare.

WHAT ARE THE RISK FACTORS FOR GLAUCOMA?

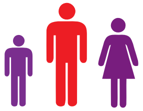

AGE

Glaucoma becomes more likely as you get older (over 40).

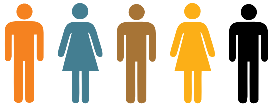

ETHNICITY

People of African, Afro-Caribbean and Asian origin, have a greater chance of getting glaucoma.

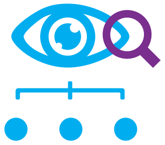

FAMILY HISTORY

If you have a close relative, such as a parent, brother, or sister who has glaucoma, you may also have an increased chance of getting glaucoma yourself.

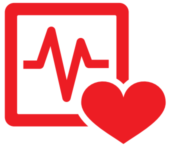

MEDICAL HISTORY

If you have diabetes or high bloodpressure, you are at more risk of getting glaucoma.

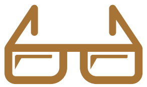

SHORT-SIGHTEDNESS

People who are short-sighted are more at risk of getting glaucoma.

If you are diagnosed with glaucoma your close relatives should be encouraged to visit an optometrist for an eye examination, as they are at increased risk of developing glaucoma. In Scotland the cost of this eye examination is covered by the NHS so it will be free for your relative to have it.

"Following diagnosis I discovered that it was a hereditary disease and that all the family knew about our risk except me. My brother and my sister had been having regular tests for some time". **Roy, age 78**

WHAT CAN I DO TO HELP MYSELF?

Have your eyes tested regularly

Glaucoma is often picked up by a routine eye test so you should have your eyes checked regularly. When you have an eye test, your optometrist will check your sight and will look for signs of eye disease such as glaucoma. You should have the routine tests described in the table on page 10.

The cost of an eye test is covered by the NHS so it is free when you have it.

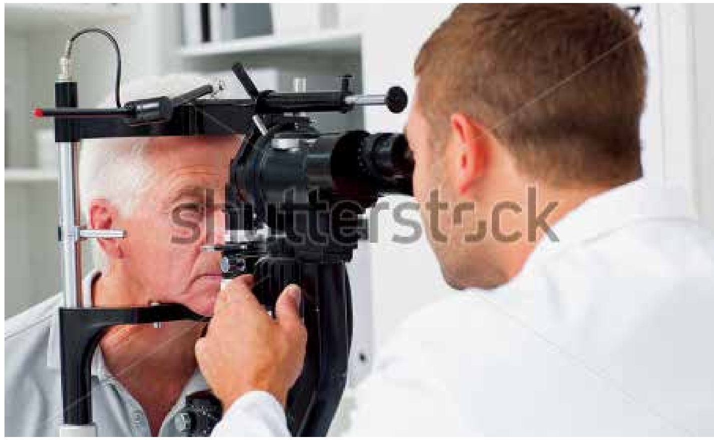

A piece of equipment that illuminates structures of your eye and then magnifies them to allow your optometrists to view the structures in detail

Don't delay act immediately.
I was diagnosed with Glaucoma at the age of 62 following some months of thinking that I needed new glasses but was always too busy to go to the opticians. The pressure in both eyes was extremely high and the optician arranged a hospital appointment. **Roy, age 78**

How often should I have my eyes checked?

INCREASED EYE PRESSURE ?

If eye tests have shown that you have increased eye pressure, you should have your eyes checked **every two years** to make sure there is no glaucoma.

STRONGLY RECOMMENDED

CLOSE RELATIVE HAS GLAUCOMA?

If you have a close relative (brother, sister, mother or father, for example) who has glaucoma, you should have a review **every two years.** If you also have other risk factors (outlined on page 7) you should have your eyes checked for signs of glaucoma **every year.** You will require lifelong monitoring if you have primary angle closure and have had surgery for glaucoma.

RECOMMENDED

SHOULD I HAVE A PATIENT-HELD RECORD?

there is not enough evidence to tell us if this is of benefit. This does not mean that these have not been successful for some people.

NOT ENOUGH EVIDENCE

HOW WILL I KNOW IF I HAVE GLAUCOMA?

"I just went to optometrist for a regular eye test. I said are you sure I might have glaucoma? I was shocked". **Harry, age 76**

Routine tests

All patients who have an eye test are given three routine tests (highlighted on page #). You will have all these tests on the same day. These tests will help your optometrist to look for signs of glaucoma. These tests often need to be repeated at future visits before a diagnosis of glaucoma can be made.

Depending on the results of your tests, your optometrist will either:

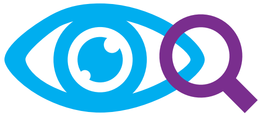

MONITOR YOU
by repeating the appropriate tests as necessary;

OR

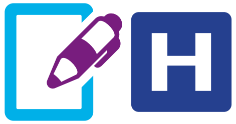

REFER YOU
to a hospital eye specialist (ophthalmologist).

Routine tests STRONGLY RECOMMENDED based on the research evidence

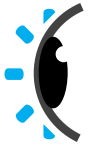

AN INTRAOCULAR PRESSURE TEST

Your optometrist blows a gentle puff of air across the surface of your open eye.

An instrument called a tonometer measures the eye's resistance to the air, and calculates your eye pressure.

Your optometrist should do **at least two** eye pressure tests using the same tonometer.

HIGHLY RECOMMENDED

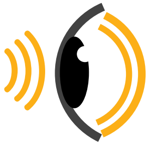

AN OPTIC DISC APPEARANCE TEST

Uses a special torch and magnifier to look at the optic nerve at the back of your eye.

If you have suspected glaucoma your optic discs should be assessed using a slit lamp (page 8). Your pupil should be enlarged (dilated) using eye drops if the optometrist thinks it is safe to do so. The appearance of your optic discs will help your optometrist decide if a referral to an eye specialist is necessary.

HIGHLY RECOMMENDED

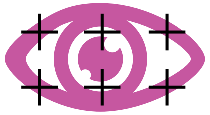

A VISUAL FIELD TEST

Checks for missing areas of vision.

Your optometrist should use a computerised machine to test your field of vision.

Your optometrist should do **at least two** visual field tests before referring you to hospital eye services.

HIGHLY RECOMMENDED

Other tests
STRONGLY RECOMMENDED
based on the research evidence

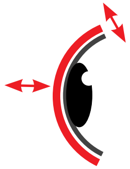

PACHYMETRY

Measures the thickness of your cornea

If you have high pressure or your optometrist thinks you might have glaucoma, the thickness of your cornea should be measured. This measurement should be stated on the referral letter when you are referred to an eye specialist.

HIGHLY RECOMMENDED

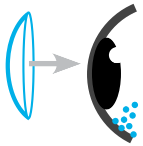

ASSESSMENT OF THE DRAINAGE AREA IN YOUR EYE (ANGLE) WHERE FLUID LEAVES IT

This helps to find out if the drainage area of your eye (called the angle) is open or closed (blocked).

When assessing the drainage area of your eye, the technique used will depend on your optometrist's preference. Techniques called slit-lamp biomicroscopy and gonioscopy are both suitable.

Slit-lamp biomicroscopy uses only a slit lamp to assess the drainage area of your eye.

Genioscopy involves placing a contact lens on the surface of your eye while you sit at the slit-lamp. You will be given anaesthetic to make this procedure comfortable.

HIGHLY RECOMMENDED

"I took my wife with me to the appointment as my letter said that I wouldn't be able to drive immediately afterwards. The drops make your vision fuzzy". **James, age 79**

GLAUCOMA: what does the SIGN guideline say?

REFERRAL TO HOSPITAL EYE CARE

WHEN SHOULD I BE REFERRED TO A HOSPITAL EYE SPECIALIST?

RECOMMENDED

IF YOUR EYE PRESSURE IS MORE THAN 25 MMHG

your optometrist may consider referring you to a hospital eye specialist. You should be referred to an eye specialist if you have one or more of the following findings from the tests on page 11:

- The appearance of your optic discs suggest that you may have glaucoma
- Vision loss
- The drainage area of your eye is at risk of becoming blocked

WHAT INFORMATION SHOULD MY OPTOMETRIST OFFER ME WHEN REFERRING ME TO A HOSPITAL EYE SPECIALIST?

RECOMMENDED

YOUR OPTOMETRIST SHOULD OFFER:

- Reasons for your referral to a specialist and the expected waiting times
- An explanation of suspected glaucoma and what to expect at appointment with specialist
- Reassurance that if diagnosed early and treated, you will not go blind
- Explain why you shouldn't drive to your appointment as it is likely that you will get drops in your eyes to dilate your pupils

YOUR OPTOMETRIST SHOULD EMPHASISE:

- The importance of attending the appointment
- that it might be helpful to note down any questions and concerns you may wish resolved at your appointment

Information should be in a language and format that suits you, for example a leaflet or face-to-face discussion

What information should my optometrist include in a referral letter to the eye specialist?

The referral letter should include:

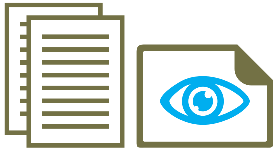

PHOTOGRAPHS AND MEASUREMENTS
of your optic discs

HIGHLY RECOMMENDED

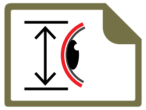

MEASUREMENTS
of thickness of your cornea

HIGHLY RECOMMENDED

It is RECOMMENDED that the referral letter should:

- Highlight the presence of any glaucoma risk factors
- Highlight the findings of the tests on page 11
- State the type of tonometer used for your eye pressure test and the time of measurement
- State the type of pachymeter used to measure corneal thickness.

GLAUCOMA: what does the SIGN guideline say?

HOSPITAL EYE CARE

What information should my eye specialist offer me?

RECOMMENDATIONS based on clinical experience	Where can I find out more?
Procedures used during eye test should be explained	**www.nhsinform.co.uk**
The importance of monitoring and treatment to preserve your sight should be made clear	**www.glaucoma-association.com**<br>**Phone: 01233 64 81 70**
Information on local sight support services	**www.rnib.org.uk**<br>**Phone: 0303 123 9999**
Advice on rights and responsibilities in line with current DVLA requirements should be given	**www.glaucoma-association.com**<br>**Phone: 01233 64 81 70**
The Certificate of Blindness or Defective vision should be explained as appropriate	**www.gov.uk/glaucoma-and-driving**<br>**Phone: 0300 790 6806**
Face-to-face and written information should be offered<br><br>You should be made aware that glaucoma runs in families and that your close family members over the age of 40 might wish have eye tests.<br><br>You should be offered the opportunity to ask questions	**www.rnib.org.uk**<br><br>**www.glaucoma-association.com**<br>**Phone: 01233 64 81 70**

Turn to **page 19** for details of organisations who can help.

"I knew nothing about glaucoma. I got a leaflet given to me and it helped me to understand it"
Nancy, age 69

DISCHARGE FROM HOSPITAL EYE CARE

The majority of patients with glaucoma will be monitored by hospital eye care services. Sometimes, it may be appropriate for people to have follow-up appointments with an appropriately qualified optometrist out with the hospital instead (see the table below). If it is appropriate for you, the hospital specialist will discuss this with you and may discharge you to the care of an appropriately qualified optometrist.

RECOMMENDATIONS
based on clinical experience

Your eye pressure is at a level that does not need treatment and your eyes on examination are otherwise normal

Your eye pressure is at a level that does not need treatment. Your eyes on examination are otherwise normal and you are at low risk of vision loss caused by glaucoma

Your eye pressure is at a level that needs treatment and is now stable. It is possible for you to be monitored through follow up appointments with your optometrist

You have primary angle closure after surgery to your eye. You are not on any medication and there is no sign of glaucoma

You are being treated for glaucoma which is working well. After discussion with your hospital eye specialist, have decided that it is best for you, at the moment, to attend a local optometrist with special skills in glaucoma.

WHAT INFORMATION SHOULD BE IN MY DISCHARGE LETTER?

RECOMMENDATIONS
based on clinical experience

Identifying information about you, such as your date of birth and address

Details about your condition including the type of glaucoma and results of the latest tests outlined on page 11

Information about your medication

Information on allergies or reactions to medication

Instructions on reasons to refer you back to the eye specialist, for example defined eye pressure

Contact details for re-referral to eye specialist in case you are returned to hospital eye services again

A copy of your discharge letter should be sent to your optometrist and your GP. It is useful for you to have a copy of your discharge letter in case you visit a different optometrists in the future.

"You can imagine my shock when one day my consultant said to me that maybe I shouldn't be driving"
Derek, age 65

INFORMATION
that might be helpful

You should be offered:

- ☐ written information on glaucoma
- ☐ instructions on when and how to use your medication
- ☐ an explanation of the importance of eye hygiene
- ☐ the opportunity to discuss the side effects from any medication and how your medication should be stored
- ☐ explanation of the issues of driving when you have glaucoma
- ☐ a clear explanation of the importance of attending follow-up appointments

You can record information about your medication in this space.

INFORMATION
Where can I get more help?

IGA – International Glaucoma Association
Woodcote House,
15 Highpoint Business Village
Henwood, Ashford
Kent TN24 8DH
Phone: 01233648179
Helpline: 01233 648 170
(open Monday to Friday from 9:30am – 5:00pm)
E-mail: info@iga.org.uk
www.glaucoma-association.com
IGA is a UK charity which works to prevent glaucoma blindness by providing information, literature, advice and support groups. Details of support groups in Scotland can be found by contacting the helpline.

Driver and vehicle licensing agency (DVLA)
Phone: 030079068806
www.gov.uk/driving-medical-conditions
The DVLA issues driving licences and keeps a database of drivers and vehicles. It has information on driving when you have a medical condition.

NHS Inform
www.nhsinform.co.uk
NHS Inform provides quality-assured health information for the public.

Sightline
www.sightlinedirectory.org.uk
Sightline is an online directory of services and organisations that help blind and partially sighted people in the UK.

Royal College of Opthalmologists
This organisation produces a range of patient booklets which may be downloaded.
www.rcophth.ac.uk

Royal National Institute of Blind People (RNIB)
Phone: 0303 123 9999
Email: helpline@rnib.org.uk
RNIB provides practical and emotional support for people affected by sight loss.

WHAT IS SIGN?

The Scottish Intercollegiate Guideline Network (SIGN) writes guidelines which give advice for healthcare professionals, patients and carers about the best treatments that are available. We write these guidelines by working with healthcare professionals, other NHS staff, patients, carers and members of the public.

How are SIGN guidelines produced?

1. Identify questions
2. Search for evidence
3. Look at the evidence
4. Make judgements & recommendations
5. Publish recommendations
6. Let everybody know about our guidelines

Our guidelines are based on the most up-to-date scientific evidence. We read research papers to find evidence for the best way to diagnose, treat and care for patients. If the evidence is not clear, healthcare professionals use their clinical experience and judgement to suggest treatments.

You can read more about us by visiting **www.sign.ac.uk** or you can phone **0131 623 4720** and ask for a copy of our booklet *'SIGN guidelines: information for patients, carers and the public'.*

If you would like a copy of this booklet in another language or format such as in large print, please phone Karen Graham, Patient Involvement Officer, on **0131 623 4740,** or email her at **karen.graham2@nhs.net.**

www.sign.ac.uk

Healthcare Improvement Scotland

www.healthcareimprovementscotland.org

Edinburgh Office | Gyle Square | 1 South Gyle Crescent | Edinburgh EH12 9E
Telephone 0131 623 4300 Fax 0131 623 4299

Glasgow Office | Delta House | 50 West Nile Street | Glasgow | G1 2NP
Telephone 0141 225 6999 Fax 0141 248 3776

The Healthcare Environment Inspectorate, the Scottish Health Council, the Scottish Health Technologies Group, the Scottish Intercollegiate Guidelines Network (SIGN) and the Scottish Medicines Consortium are key components of our organisation.

HEI Healthcare Environment Inspectorate
Ensuring your hospital is **safe** and **clean**

scottish health council
making sure your voice counts

SIGN

Scottish Health Technologies Group
