# Supplementary material for: Improving the user experience of patient versions of clinical guidelines: user testing of a Scottish Intercollegiate Guideline Network (SIGN) patient version
Source: BMC Health Serv Res. 2016 Feb 2;16:37. doi: 10.1186/s12913-016-1287-8 (PMC4736267; doi:10.1186/s12913-016-1287-8)

STROKE RISK

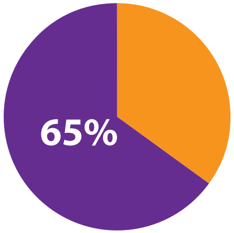

65% PEOPLE
WILL NOT HAVE A STROKE WHETHER THEY TAKE WARFARIN OR NOT

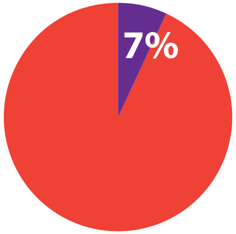

7% PEOPLE
WILL BE SAVED FROM HAVING A STROKE BY TAKING ASPIRIN

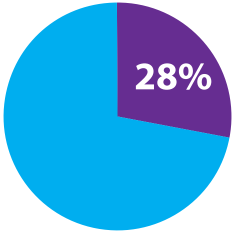

28% PEOPLE
WILL STILL HAVE A STROKE EVEN THOUGH THEY TOOK ASPIRIN

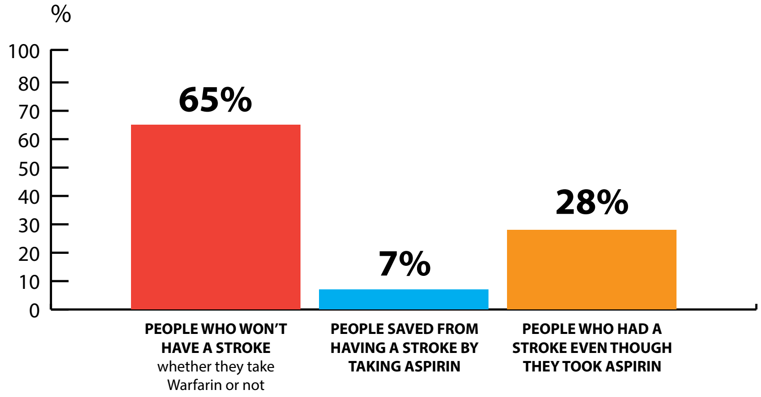

Supplement: Supplementary file 3 — Supplementary bar and pie charts. (PDF 144 kb) [file 12913_2016_1287_MOESM3_ESM.pdf]
