# Supplementary material for: Improving the user experience of patient versions of clinical guidelines: user testing of a Scottish Intercollegiate Guideline Network (SIGN) patient version
Source: BMC Health Serv Res. 2016 Feb 2;16:37. doi: 10.1186/s12913-016-1287-8 (PMC4736267; doi:10.1186/s12913-016-1287-8)
Supplement: Supplementary file 4 — Table of changes made to the draft SIGN guideline following user testing. (PDF 283 kb) [file 12913_2016_1287_MOESM4_ESM.pdf]

Summary of Comments on SIGN Glaucoma – User Testing

Page	Sequence Number	Comment	Action
Front cover	1	General changes Font size/booklet size: Needs to be in a font that is dark and large enough for people with some sight difficulties to read.<br><br>Colour: Use of light colours makes difficult to read e.g. light blue/yellow/orange - can these be replaced with darker colours and more black text.<br><br>Pages cramped: Need to be spaced out more. Feedback is the less on the page the better really.<br><br>One person noted the use of centred text on all the paragraphs and would have preferred standard left style - should this be changed at least for the main paragraphs?	Changed.
	2	Testing suggests the line "what does the SIGN guideline say" does not convey any information because of low awareness of SIGN. Should it be replaced e.g. Referral and safe discharge - or something more informal like "Am I at risk? Getting diagnosed and getting referred."	Completed with SIGN style.
	3	Image needs to change - very strong negative feedback re the emotional tone and lack of link to glaucoma. What people want is an image that links to glaucoma or at least directly to the eye.	Changed.
Contents page	1	If title is changed these need to change throughout. Too small for most people to read at the moment.	Taken out.
	2	Can title and headings be in large bold text but NOT all capitals throughout	Taken capitals out.
Contents page (contd)	3	Several people have mentioned that black bold text would be clearer - here is readable because of the size but elsewhere black would be easier.	Kept top heading and some dark coloured for variety and style.
	4	Two people mention the lack of page number references here like p.2	Page numbers in contents page reverted to traditional style.

	5	Your Eye - page 4 and 5 is missing.	Changed.
	6	Is page 9 missing How often should I have my eyes checked? - or is it a continuation of What can I do to help myself?	Made subheading clearer.
	7	This page confused the testers because they thought the leaflet would go to people that have already been diagnosed. Hopefully clearer if title changed as suggested.	Title changed.
	8	Page numbers are too small for people to read. Preference would be for a larger, bold, black font.	
2 & 3	1	Some people found the tone of this bit aggressive/prescriptive. Maybe too many capitals and quite a lot of red doesn't help.<br><br>Some people felt it could be confusing as to which group you belonged to unless a clinician has given this to you and ticked the right one.	Changed heading page 2.
	2	Title doesn't capture carers/friends currently. Would it be worth considering softening tone a bit and having a heading more like "who is this booklet for"	Changed.
	3	Amend to clarify.<br><br>I realise getting diagnosed may be confusing for at risk group and isn't quite the same as referral but may make more sense to the readers - they found this whole section bureaucratic in tone.<br><br>Maybe give SIGN in full on page 20 adding to bureaucratic tone here.	Guideline did not cover diagnosis, so did not change to this wording BUT reworded to give information first, then refer to page 24 for SIGN Information.<br><br>Need the first use to be in full, so people understand who we are.
	7	Danger that this icon can be confused with a 1 in 5 risk – which it does not mean to convey. Could it be replaced with something indicating high instead?<br><br>Note in the last test material, one person mistook the blue circle used for diabetes as a zero indicating zero risk so	Designer considered both. We agreed that there was no other concise way to convey this, changed red to green.<br><br>Not in this guideline.

		needs to be careful with icons.	
	9	Can this have a snappier title that people may be more likely to read like: Strength of Recommendation, and give this bit of info in the paragraph above.	Used "Types" to simplify. Covered in restructure of paragraph.
	10	Use of colour here is not quite right. This was red because it initially said do NOT do xxx. Now it should be a neutral colour like amber or stick with them all being green. Several people said red looks like something they should not do.	Changed.
	11	The bit underneath each one in black needs to stand out way more e.g. bold.<br><br>Testers did not pick up on one being research based and one being clinical experience – they just considered it to reflect how important we thought they were.<br><br>Also, consider leaving this text on the page with the recommendations.	Addressed by restructuring paragraph.<br><br>Rejected due to cluttering and duplication.
	12	This was considered key information that should be higher up the page – if you remove the second highlighted paragraph it could be moved to above the symbols.<br><br>Also, consider leaving this text on the page with the recommendations.	Rejected due to cluttering and duplication.
	14	This is very important so needs to be made to stand out in bold or as first line on this page. Most people missed it and then asked why no information on treatment. Having a clearer title should also help.	Changed.
2 & 3 (contd)	15	Some queries about why this is here given contents on the previous page - at the moment too small and squashed for people to use. Remove and spread out the rest of the page?	Gives information in different ways and reminds people, so retained.
	16	Why does just this one bullet point have a semi colon error?	Changed.

	17	People want a phone number or address as well. Could this be moved onto page 20 where the phone number is given?	There is always discussion about where the information about SIGN goes:<br>• some reviewers say at the beginning<br>• some reviewers say at the back<br>• happy medium – the contact details are given in the about SIGN section
4 & 5	1	People were very positive about this diagram only comments were if it is possible to slightly increase the font size of the words.<br>Is it possible to fill the page with the diagram - increase font size of the title?	Already as large as possible due to the constraints of image and design.
	2	Here and on previous page use a non capital style of heading - this should be consistent throughout - I would say in this style but a larger font.	Changed.
	3	Can all this be in black text except for the NORMAL and HIGHER below which probably work in colour.	Changed.
	4	MMHG being in capitals here is read as an error by people that know chemical notation and expect it to be mmHg. It would be better to use non capitals in a large font here.	Changed.
6 & 7	1	Feedback is this page is too cluttered with the paragraph and quote below. May need the quote to come out or the paragraph/ quote to go somewhere else.	Altered to be over 2 pages.
	2	This doesn’t really give people enough information They want to know that it is a lot more common in people over age xx as well. Instead of over 40 most common in 5th to 7th decade of life or similar.<br>They also notice that the icon doesn't really have anything to do with age.	“in 5th to 7th decade” - this level of detail is not in the guideline of plain English.<br>Tried but difficult not to get stereotypical.
6 & 7 (contd)	3	Overall most people were quite keen on including the 2 in 100 information in the booklet if clearly linked to getting your eyes checked. So if it was to go in this bit on risk would need its own page. If it does go in it is important to	2 in 100 was taken out of the guideline, as the group was not happy on its level of accuracy.

		get in that the risk gets higher as you get older and some more contextual information. some people thought 2 in 100 was high and some people thought it was low! Also important if it goes in it is linked to an action such as having your eyes tested.	
	4	The sentence "If it is diagnosed and treated early enough, further damage to your sight can be prevented. Usually, people have to have treatment for glaucoma for the rest of their lives" was considered very important and should be highlighted in some way.<br>Testers were keen on more direct encouragement of people to get their eyes checked.	Changed (x2).
	5	One tester didn't like use of word blind since very scary would prefer visually impairment or something similar.	Retained as conveys the seriousness of the message.
	7	Testers want a direct link between risks and stats and something to do about it - so may need a line about having regular eye test or reference to page with recs on here.	Changed.
	8	Move boxes to beneath the first paragraph.<br>Mixed feedback on these boxes - some people do not like the different sizes and others liked the boxes. Could it be kept in boxes but all made the same size?	Changed BUT felt this was perhaps more the personal preference of this particular patient group.
	9	Red may best be avoided since people associated it with something bad	Changed.
	10	The testers considered both these things to be very important and they should be emphasised more they are getting lost on this page and in this small font. Suggestion was that this info fits with the recommendations on page 9.<br>Also add in that you can get the free eye test from your local optician.	Spread out over several pages.
8 & 9	1	First heading needs to be up here so the space can be used better.	Page restructured to take this into account.

		Fonts are too small and could be spaced out better. I would suggest just having the first two recs on this page with the extra paragraph and/or quote from page 7.	
	2	It may not have been clear to people that these recommendations were for people that do not have a diagnosis of glaucoma yet. Maybe needs to be added into title.	Did not change, as felt this was clear from the sequence of the guideline.
	3	Heading doesn't match the third recommendation - so it either needs a separate page or a new heading.	Restructured page.
	4	People could not understand why these said every two years when they had annual eye tests.	The guideline is based on evidence, not on current practice – practice should change in future.
	5	People don't pick up on second sentence they only read the first. Separate in some way, maybe two bullet points.	Changed.
	6	Users suggested that this be emphasised in bold or with some kind of symbol like an exclamation mark to emphasise its importance.<br><br>They also think the link between not getting your eyes tested and potential loss of sight needs to be emphasised somewhere. Also that people do not necessarily notice anything wrong with their eye sight - so need this explaining.	Exclamation mark is does not fit with house style.<br><br>Agree – supported by the patient quote that it is the optician's role to explain.
	8	Delete highlighted text - multiple people suggest it doesn't appear to relate to the rest of the information.	Agree – changed.
	9	Consider keeping in the based on research evidence under the strongly recommended symbol (like on page 3) and the based on clinical expertise under the recommended etc since people just don't pick up on the difference at the moment?<br><br>This may help to clear up why some are strong and some not.	Did not change to avoid cluttering and duplication.
	10	This recommendation should either be removed or given a page of its own. Testers do not know what one is so	Changed (x2).

		cannot make sense of it. They are not sure what they would do anyway with the message of uncertainty. Slight change to text may help this.<br><br>If it stays in it needs an accompanying explanation of what a patient held record is. One concern raised by multiple people is the belief that they would hold the only record and fear losing it.	
8 & 9	12	Double negative in this sentence makes it hard to understand. Amend: e.g. "Some people may find having one helpful, but other people may not.	Changed.
	13	Could this be changed to something like "We don't know yet" not sure but it avoids using the term evidence. Since people find it confusing, not coming from their perspective, they are not clear on what counts as evidence and what doesn't. Consider that if it is in use that is evidence or that there must be evidence.<br><br>But basically the symbol works to convey uncertainty that is the message that people get, they just don’t like it being uncertain. So it may be no change is necessary.	Changed and added research too.
	14	If kept in colour needs to change from red to green or neutral colour like amber.	Agreed.

Summary of Comments on SIGN Glaucoma – User Testing

10 & 11	1	Does the strongly recommended need to be here and under each test?<br><br>Could this be replaced with "given as part of a normal eye check up" or similar. It may not have been that clear that this was part of a routine eye test.<br><br>The style of the headings changes quite a lot - I think people only see the strongly recommend here because it stands out so much and they miss the rest of the text - can it just all be black bold non capitals.	Restructured to take away duplication and make the connection with the heading better.
	2	Orange text too light - consider change to black.	Changed.
	3	This blue colour is hard to see and needs to be replaced with something darker - maybe just black text, keeping the colour in the icon only.	Changed.
	4	It doesn't identify the image as of a slit lamp on page 8 needs to be added in.	Changed.
	8	All ones with this much text didn't seem to get read. Does all this need to be in. Could we delete the second sentence and just change the second one to "Your pupil may be enlarged using eye drops".	Agreed.
	9	All highly recommended needs to be changed to strongly recommended as written on the guide on page 3. Which is all from now on.	Changed.
	10	Pink text too light consider change to black	Changed.
	11	Some testers suggest that a page that tells you the difference between an opthalmologist, optician and optometrist would be very helpful.	Patients involved in the development of the text did not think this was necessary. Opthamlologist was explained in the text.
12 & 13	1	Testers suggested this page is cramped and should be spread over two pages - you could spread over two and shift a quote or something in as well.	Restructured to make better use of space.
	2	Does this need strongly recommended here and under each recommendation.	Did not change – see above.

Summary of Comments on SIGN Glaucoma – User Testing

12 & 13	3	The light blue and orange is hard to read. Could keep the boxes coloured and make the text black.	Agreed.
	4	Quite a few testers had been referred to a specialist by their GP not their optometrist and found this confusing – does this need to be acknowledged?	No – doesn't often happen and not what would be expected.
	5	Need to direct to page with the explanation of mmHg on - since most people have no idea what this is. Another suggestion by some testers that here it could just say high.<br><br>Could we make the referral back to page using icons e.g. in a little speech bubble or using a symbol like an exclamation mark?	Did not think anything to be gained by giving explanation again on this page. There is an explanation on another page.<br><br>For consideration in future guidelines.
	6	I think this confused people because the line in blue looks like a heading but the bits beneath are different areas not just for people with high eye pressure.	Made headings more consistent.
	9	The language on this page was considered too technical by quite a few people although some people also appreciated the information.<br><br>In particular "slit-lamp biomicroscopy and gonioscopy" and so on considered "blinding people with science".<br><br>One suggestion was heading with the function of the test	Agreed. Changes made to headings and technical details in text to help.
	10	This colour is too light consider changing both headings on this page to black text.	Agreed.
	13	Consider removing the technical terms and just leaving in the information.<br><br>One suggestion was heading with the purpose of the test e.g. the bit currently in bold black text and giving the more technical bits underneath.	Changes made to headings and technical details in text to help – there is always a discussion about the level of technical detail, try to find middle ground.
	15	One person wasn't clear on if this is waiting in a waiting room or waiting time to see the specialist - is it worth	Did not change. This is an individual's experience. The text was clear to most.

Summary of Comments on SIGN Glaucoma – User Testing

		spelling it out?	
	16	About instead of an explanation of.	Changed bullets.
	18	People identify this (encouragement to access treatment early etc) as key along with the importance of attending your appointment below - can they be emphasised in some way e.g. bold make first bullet.	In this order to emphasise what came out in the guideline development about important patient issues.
	20	Highlighted text considered over wordy by some testers - consider change.	Did not change. Considered a style issue – we were happy with it as written.
	21	People really liked this but did specifically interpret it as meaning that people would always be able to access material in their own language - is that true?	Yes – legal obligation.
14 & 15	1	Does this line just repeat the title above?	Left this as it is for clarity and readability.
	2	It may be that black text here would be easier to read.	Changed.
	3	Full stop here and not on other sentences	Changed
	4	This needs to be in a darker text – probably just black – larger font throughout, but clearly required on this page.	Agreed.
16 & 17	1	Highlighted text may be a bit wordy - has appropriate in it a lot. Could it be simplified a bit e.g. "Some people may have follow-up appointments with an optometrist who is not based in the hospital. If this is right for you, the hospital specialist will talk with you about it and discharge you to the care of a suitable optometrist.	Agreed.
	3	This table needs a heading. I find it a bit unclear what it is talking about. Is it "when you are suitable for discharge" Or "When you are suitable for discharge to care by an optometrist in the community" or something similar?	Agreed.
16 & 17	4	"Instructions on reasons to refer you back to the eye specialist, for example defined eye pressure"<br>When instead of reason? Or does that change the meaning?	Why and when to clarify.

	6	Testers indicated that you are returned sounds a bit like you are a package! What about "...in case you need to go back to hospital eye services again"	Agreed. PEC
	7	Needs to be made more prominent with font size etc.	Agreed.
	8	We have had one person raise concerns that passing the information on about clinicians stopping you from driving may put people off having their eyes checked.<br>Other people talked about being shocked by this when they saw the clinician and were told abruptly they wouldn't be able to drive. On balance may be best to leave it in - but would be good if SIGN could think about whether any more info on this should go in considering how important to the testers it was.	Mentioned in a few places.<br>Not SIGN's remit to give information.
18 & 19	1	Is it worth adding something in like NHS24 or something re mention of need for a medical contact for emergencies/inquiries?<br>Not sure if that is an appropriate contact for non emergency inquiries but there is mention of need for something like this.<br>The other group mentioned by several people is NICE - not sure if just confusing to give them as a source of information as well as SIGN? But they do have the guideline on treatment which is what is missing from here?!	Did not change. Not an emergency situation.<br>Did not change. Worried that this would confuse people.
	2	Language here could be simplified e.g. "A chance to talk about...."	Changed.
	3	"an" needs to be added before explanation as the bullet above.	Changed.
		Could it just be "information about driving and glaucoma" or "a discussion about driving and glaucoma"	
20	1	One tester suggested having the SIGN logo on this page again to make the association clear.	Good idea, but would cramp page.

Summary of Comments on SIGN Glaucoma – User Testing

	2	Patients are interested in the status of these guidelines in terms of whether they can be used to make services give them a certain treatment - whether these are standards of care that have to be adhered to. - Not sure that this can easily be tackled or explained here.	Did not change – more appropriate to search for another website if interested. Also not everyone wants to know.
	3	The reference to public involvement here doesn't seem to stand out enough. Some testers wondered where patients/public are involved and didn't see it here.<br><br>Can a line or something on public involvement be added in?	Agree, but did not change because SIGN would need more time to work on diagram to include MDG.
	4	Need a bolder heading and as suggested below maybe a separate heading pages about clinical guidelines - since some users just didn't get that this showed the process of making a guideline.	Changed top heading.
	5	The colours used for the first three icons are too light for the readers to see easily. Text below each too small - as it is throughout the booklet.	Agreed - changed.
	6	One tester asked specifically for this to be changed to mostly ticks since having three crosses is too negative.	Did not change. This reflects that we reject more than we use, i.e. reflects quality.
	7	Testers thought that this paragraph was the key information and should be at the top of the page.<br><br>"Our guidelines are based on the most up-to-date scientific evidence. We read research papers to find evidence for the best way to diagnose, treat and care for patients. If the evidence is not clear, healthcare professionals use their clinical experience and judgement to suggest treatments."<br><br>Perhaps move to top and head "Our guidelines" or "What is a SIGN guideline? With this paragraph and the icons and have what is SIGN and how to order the booklet on another page?	Not for this guideline, as pages layout worked well.
	8	People uncertain what we mean by evidence here - could it be changed to something like "If we cannot find this out	Agreed.

		from the research evidence, we ask healthcare professionals to use their clinical experience and judgment”	
	9	Suggestion to give contact details altogether once here rather than in the two paragraphs.	Not changed. Too different purposes and contact numbers.
